# The D_3_ ‐creatine dilution method non‐invasively measures muscle mass in mice

**DOI:** 10.1111/acel.13897

**Published:** 2023-06-05

**Authors:** Lauren Wimer, Elena Goncharova, Sofiya Galkina, Edna Nyangau, Mahalakshmi Shankaran, Asia Davis, Leandro Prado, Maria Castro Munoz, Sharon Epstein, Cavan Patterson, Nicholas Shaum, Mark Hellerstein, William Evans, Simon Melov

**Affiliations:** ^1^ Buck Institute for Research on Aging Novato California USA; ^2^ Department of Nutritional Sciences and Toxicology University of California Berkeley California USA; ^3^ Astera Institute Berkeley California USA

**Keywords:** aging, mice, sarcopenia, skeletal muscle

## Abstract

Developing accurate methods to quantify age‐related muscle loss (sarcopenia) could greatly accelerate development of therapies to treat muscle loss in the elderly, as current methods are inaccurate or expensive. The current gold standard method for quantifying sarcopenia is dual‐energy X‐ray absorptiometry (DXA) but does not measure muscle directly—it is a composite measure quantifying “lean mass” (muscle) excluding fat and bone. In humans, DXA overestimates muscle mass, which has led to erroneous conclusions about the importance of skeletal muscle in human health and disease. In animal models, DXA is a popular method for measuring lean mass. However, instrumentation is expensive and is potentially limited by anesthesia concerns. Recently, the D_3_‐creatine (D_3_Cr) dilution method for quantifying muscle mass was developed in humans and rats. This method is faster, cheaper, and more accurate than DXA. Here, we demonstrate that the D_3_Cr method is a specific assay for muscle mass in mice, and we test associations with DXA and body weight. We evaluated the D_3_Cr method compared to DXA‐determined lean body mass (LBM) in aged mice and reported that DXA consistently overestimates muscle mass with age. Overall, we provide evidence that the D_3_Cr dilution method directly measures muscle mass in mice. Combined with its ease of use, accessibility, and non‐invasive nature, the method may prove to more quickly advance development of preclinical therapies targeting sarcopenia.

## Graphical abstract

We demonstrate that the D3‐creatine dilution method can be used to measure skeletal muscle mass in previously unexplored murine model, *Mus musculus*. We evaluated the results from the D_3_Cr method with DXA‐determined lean mass in mice and reported that DXA consistently overestimates muscle mass with age. Overall, we provide evidence that the D_3_Cr method measures skeletal muscle mass; combined with its ease of use, accessibility, and non‐invasive nature, the method may prove to advance development of preclinical therapies targeting sarcopenia.

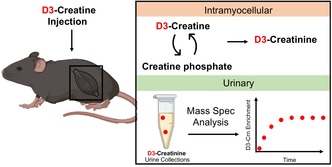

**Abbreviations**
BM: bone mass
CT: computed tomography
D3Cr: D3‐Creatine
DXA: dual x‐ray absorptiometry
FBM: fat body mass
LBM: lean body mass
MM: muscle mass
MRI: magnetic resonance imaging

## 1 INTRODUCTION

Skeletal muscle makes up the largest fraction of tissue in the body (Wang et al., 1992), though the accurate quantification of skeletal muscle mass remains elusive. Accurate quantitation of skeletal muscle is vital for studying sarcopenia, an age‐associated syndrome defined as the gradual loss of muscle mass resulting in functional decline, disability, and loss of independence. Quantification of muscle size with imaging using computed tomography (CT) and magnetic resonance imaging (MRI) is available; however, these methods are expensive, slow, and impractical in large cohort studies. Both methods are powerful tools for the characterization of body composition, though they have drawbacks such as ionizing radiation and necessary special expertise to infer muscle mass (Meganck & Liu, 2017). These limitations provide a rationale for alternative muscle mass quantification methods.

Dual x‐ray absorptiometry (DXA) has emerged as the most commonly used method for body composition analysis. DXA uses a three‐compartment categorization method: Bone and fat mass are separately quantified through the differential absorption of high and low photon energies, and then, lean mass (all remaining tissues excluding fat and bone) is differentially calculated. The method's popularity results from its relatively quick and easy measurement, low radiation dose, and precision. However, its use is often limited due to instrumentation availability and cost. Although DXA is used as a surrogate for muscle, it does not measure muscle mass.

A method for the non‐invasive measurement of muscle mass was recently reported (Clark et al., 2014). The D_3_‐creatine (D_3_Cr) dilution method is non‐invasive, accurate, and has the potential to become the new gold standard for quantifying muscle mass in preclinical models (Stimpson et al., 2012). Isotopically labeled creatine had been previously employed to determine creatine pool size in humans, with positive correlations to functional capacity and risk of injurious falls disability, and mortality (Cawthon et al., 2019, Cawthon et al., 2021, Cawthon et al., 2022). This correlation is noticeably absent in the literature of similar studies using DXA (Schaap et al., 2013). The D_3_Cr method has also been reported to accurately measure age‐associated muscle wasting in rats (Stimpson et al., 2012) but has remained undeveloped in mice, the most commonly used preclinical model.

In our study, we assess the use of the D_3_Cr dilution method to quantify muscle mass in mice. We tested a single dosage (2 mg/kg) of methyl‐D_3_ labeled creatine and repeatedly collected urine post‐IP to determine appropriate sampling time windows. Mass spec analysis of urinary creatinine enrichment allowed for quantification of skeletal muscle mass, which was able to be directly compared with total body weight and respective DXA results.

The D3 method relies on the assumption that urine creatinine is solely derived from the total body creatine pool (about 98% of which is sequestered in the sarcomere) (Clark et al., 2014). To accurately measure creatine and creatinine ratios (Cr/Crn) in urine, it is important to identify all potential exogenous sources of these metabolites, as dietary creatinine can confound the quantification of urine creatinine enrichment with deuterium. Mice in our study were all purchased from Jackson Laboratories and reared on a low‐fat diet (Envigo Teklad #2918) free of animal products or potential dietary Cr/Crn. Therefore, all Crn detected in urine of mice in this study is derived from endogenous production or administered D_3_Cr, primarily metabolized in muscle.

**FIGURE 1:**
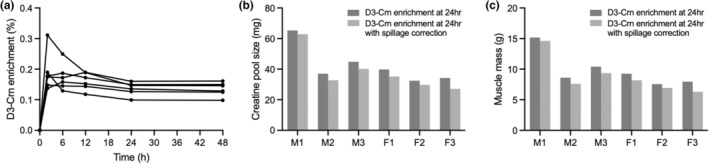
Creatine pool size and muscle mass can be calculated from mouse urine by D3‐creatinine enrichment following an IP injection of D3‐labeled creatine. (a) D_3_‐creatinine enrichment values at varying timepoints from individual urine samples of young mice following an IP injection of D_3_‐labeled creatine. (b) Creatine pool size (mg) values of individual mice calculated using their respective D_3_‐creatinine enrichment at 24 h. Bars represent creatine pool sizes either uncorrected or corrected with spillage effects observed at 0, 2, 6, and, 12 h post‐IP. (c) Muscle mass (g) values of individual mice calculated using their respective D3‐creatinine enrichment at 24 h. Bars represent creatine pool sizes either uncorrected or corrected with spillage effects observed at 0, 2, 6, and, 12 h post‐IP. “M#” individual male mice, “F#” individual female mice.

To determine whether our dosage of D_3_Cr was sufficient to measure muscle mass, we carried out a pilot study in young C57BL/6J mice (5 months old, males and females, Jackson Laboratories #000664). These initial studies used a single intraperitoneal (IP) injection of D_3_‐creatine at 2 mg/kg (mpk) of body weight. This dosage was chosen based on prior studies that mice demonstrate increased creatinuria and findings that higher doses (>4mpk) of injected creatine were necessary to elevate plasma creatine levels (AA & AE, 1958). Unfasted mice received a single injection of D_3_Cr, and urine was collected at 0, 2, 6, 12, 24, and 48 h post‐IP. Sequential urine collections were used to determine the optimal window to capture urinary D_3_Crn enrichment, which reflects the percentage of converted D_3_‐labeled creatine to creatinine in the total creatine pool. Similar to previous reports in rats (Stimpson et al., 2012), urinary creatinine enrichment was highest at 6 h post‐IP, and steady state enrichment was achieved by 24 h (Figure 1a). Steady state enrichment was still observed at 48 h, though it is undetermined within our study how long this steady state may last in mice. Consistent with prior literature, we assume that once steady state enrichment is achieved, the enrichment of urine D_3−_creatinine will match the enrichment of intramyocellular D_3_Cr as a result of the irreversible conversion of creatine to creatinine, reported to be ~2% per day (Heymsfield et al., 1983).

Total creatine pool size of each pilot sample was calculated from D_3_‐creatinine enrichment at 24 h post‐intraperitoneal (IP) injection (Figure 1b) using a previously reported formula for determining pool size based on the enrichment of a tracer, assuming that 98% of total body creatine is sequestered in skeletal muscle (Andrews et al., 1998). From the creatine pool size, skeletal muscle mass was derived by dividing creatine pool size by 4.3 g/kg (the average concentration of creatine in wet muscle mass (Andrews et al., 1998), Figure 1c). Both creatine pool size and muscle mass are plotted with and without spillage correction, calculated using Cr/Crn values at 2, 6, and 12 h (Shankaran et al., 2018). Due to the insubstantial contribution to total muscle mass, spillage correction was not calculated in further studies.

After deducing the appropriate sampling window, we explored whether the D_3_Cr method could be used to detect age‐associated muscle loss in a cross‐sectional analysis of mice ranging from 5 to 25 months of age. Reports in humans demonstrate that the D_3_Cr method is sufficient to observe age‐associated muscle loss (Pirker & Katzenschlager, 2017) but has been widely unexplored in murine models. As per our initial pilot studies, mice were purchased from Jackson Laboratories and reared on Cr/Crn‐free diets. Unfasted mice were subjected to a single IP injection of 2mpk body of D_3_Cr followed by urine collections at 24 h post‐IP injection. Respective body composition was determined by DXA in the same week.

**FIGURE 2:**
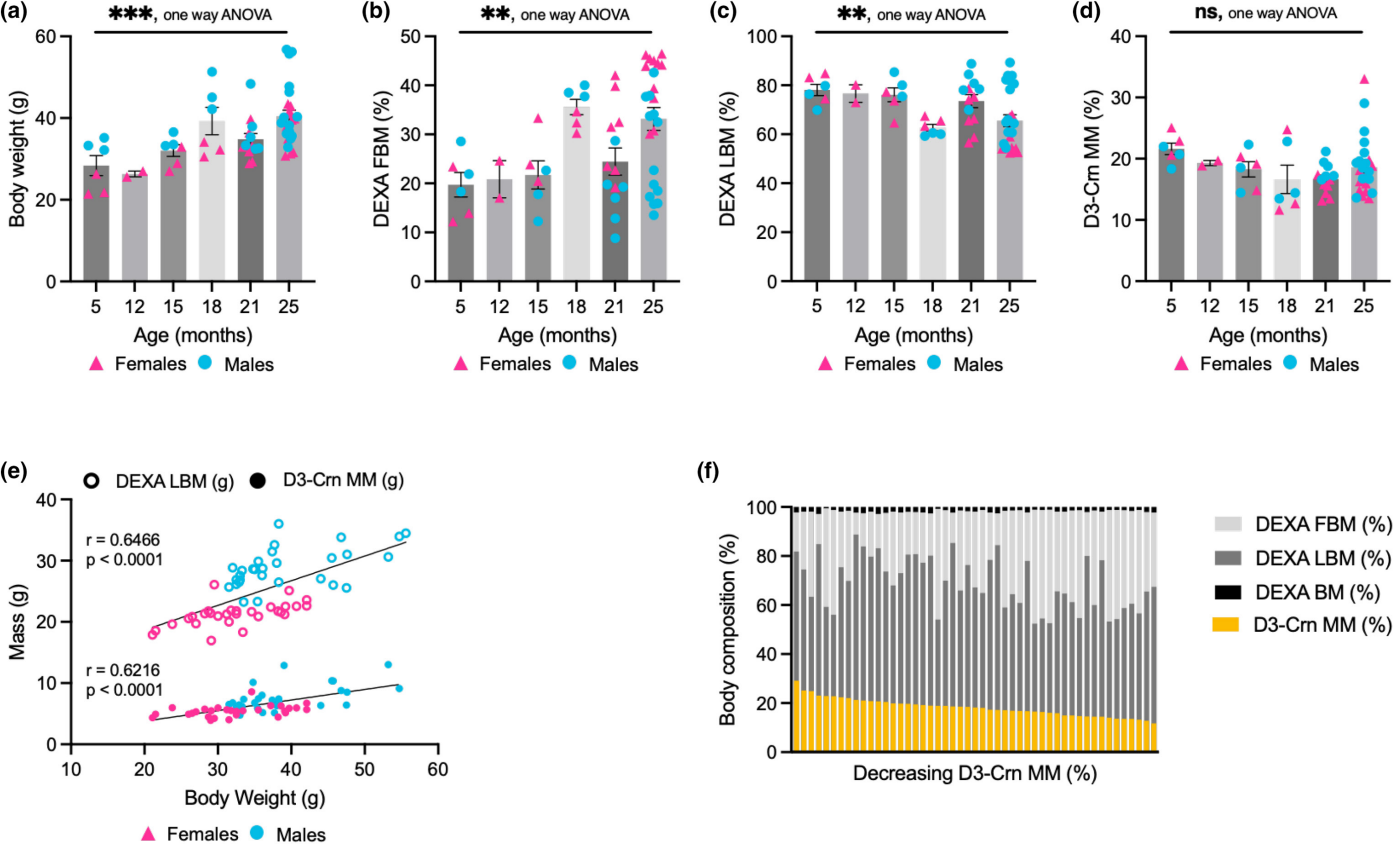
Cross‐sectional analysis of aged mice revealed lower DXA‐determined lean mass and increased fat mass independent of D_3_Cr determined skeletal muscle mass. (a) Body weights of mice, grouped by age. (b) DXA‐determined fat body mass (FBM) percentages, grouped by age. (c) DXA‐determined lean body mass (LBM) percentages, grouped by age. (d) D_3_Cr dilution method calculated muscle mass (D_3_Cr MM) percentages, grouped by age. (e) Both DXA LBM and D3‐Crn MM correlate significantly with total body weight. (f) Decreasing skeletal muscle mass determined by D3‐Crn contrasted with DEXA in mice of varying ages. BM, bone mass, FBM, fat body mass, LBM, lean body mass, D3‐Crn MM, skeletal muscle mass as determined by D_3_Cr dilution method. Significance – ns *p* > 0.05, * *p* < 0.05, **<0.005, ***<0.0005).

It is well established that mice on multiple genetic backgrounds gain weight as they age (Pappas & Nagy, 2019, Evans et al., 2021). This is unlike humans who tend to reach a plateau of total body weight while concurrently losing muscle at later ages (Alley et al., 2008). Here, we demonstrate age‐associated changes in body composition using a cross‐sectional study design with muscle quantification performed both by DXA and D_3_Cr dilution method. Consistent with prior studies in mice, we observed that mice had higher body weights with increasing age (*p* = 0.0003, one‐way ANOVA) (Figure 2a) and increased percentages of DXA‐determined fat body mass (FBM) (*p* = 0.0033, one‐way ANOVA) (Figure 2b **)**. Despite their increased total body weight, aged mice had lower lean body mass (LBM) percentages (*p* = 0.0094, one‐way ANOVA) (Figure 2c) indicating an age‐associated loss of LBM, typically attributed to muscle mass (Volpi et al., 2004). However, excluding fat, DXA cannot distinguish between non‐muscle and muscle (Shepherd et al., 2017). We explored whether the D_3_Cr method would demonstrate decreases in skeletal muscle mass, consistent with their DXA scans. Interestingly, D_3_Cr determined muscle mass (D_3_Cr MM) was not statistically altered with increasing age (5, 12, 15, 18, 21, 25 m.o., *p* = 0.1950, one‐way ANOVA) (Figure 2d).

Both DXA determined LBM and D_3_Cr muscle mass were significantly correlated with body weight (*R* ^*2*^ = 0.4181 (*p* < 0.0001), *R* ^*2*^ = 0.3864 (*p* < 0.0001)), respectively (Figure 2e), regardless of age or sex. Evaluation of LBM by DXA showed a larger total mass than D_3_Cr MM, possibly reflecting non‐muscle tissue as a component of LBM, as previously suggested. Correlation between DXA LBM and D_3_Cr MM was significant, though skewed largely by sex (Figure S1A–C); further analyses were performed by separating males and females and produced statistically insignificant results (*p* = 0.1137 and 0.5290, respectively). This finding demonstrates conflicting data for which method may be accurately determining skeletal muscle mass. Weights of individual hindlimbs muscles taken at the time of dissection were found to be statistically unchanged between age groups (Figure S1E), supporting data derived from the D_3_Cr method. Additionally, the magnitude of signal detected in both tested methods is worth emphasizing—DXA attributes 60%–80% LBM per mouse, while as D_3_Cr was ~15%–30% MM, more consistent with relative proportions of muscle mass in many mammals (Figure 2f). This suggests that the majority of signal in LBM is not derived from skeletal muscle, and the age‐associated loss being quantified may be loss of another tissue type, unexplored within our study.

Our work highlights the overestimation of DXA‐estimated muscle mass by direct comparison with D_3_Cr derived skeletal muscle mass. We demonstrate the use of the D_3_Cr dilution method in mice and apply mass spec analysis to confirm creatinine enrichment and steady state, though there are limitations to its use, and these should be considered when determining which method to use to calculate muscle mass. The D_3_Cr dilution method directly analyzes whole body muscle mass, which is made up of varying fiber types, but the formula used for the calculation of creatine pool size (and skeletal muscle mass) assumes 100% glycolytic (type II) fibers. This assumption negates differing creatine uptake rates in oxidative (type I) fibers and impedes studies in aging mice which are characterized to have altered fiber quality with age (Giacomello et al., 2020). The D_3_Cr dilution method may determine total body muscle mass but cannot reflect age‐associated changes in muscle quality. Further limitations lie in our cross‐sectional study design, as each animal undergoes individualized rates of aging. Our study design used both sexes and increasing cohort size with age, assumed to have increased variability (>21 months). Our study allowed for timely assessment of the D_3_Cr dilution method across ages, but further longitudinal studies are necessary to more accurately address the nature of sarcopenia phenotypes in aging mice. In conclusion, we demonstrate an alternative method for non‐invasively measuring total skeletal muscle mass in mice with direct comparisons to field standards. It will be important to use this methodology in tandem with other methods for future validation studies as well as measuring muscle mass in future preclinical studies of sarcopenia, utilizing longitudinal studies to better understand how muscle changes with age.

## 2 METHODS

### 2.1 D3‐creatine

D3‐creatine was purchased from Cambridge Laboratories (DLM‐1302‐0.25) (LOT#PR‐30122) (PSO#22A‐0183). Solution was made to 0.6 mg/mL in PBS for a final delivery of 2 mg/kg of body weight.

### 2.2 Mice

Studies included the use of male and virgin female C57BL/6J mice (Jackson Laboratories #000664) aged between 20 and 99 weeks of age. All mice were communally housed and aged‐matched with ad libitum access to water and diet (Envigo Teklad #2918) in a pathogen and temperature‐controlled room with a 12 h light–dark cycle beginning at 06:00 AM. All procedures were conducted in accordance with NIH Guidelines for Care and Use of Animals and were approved by the Institutional Animal Care and Use Committees at Buck Institute for Research on Aging.

Mice: Our N for young mice (5 months) was 6 (3 M/3F), for adult mice (12, 15, and 18 months) was 6 (3 M/3F), and for aged mice (21 months) was 13 (6 M/7F) and 22 (25 months) (11 M/11F), respectively.

### 2.3 Exogenous creatine

Mice were maintained on a low‐fat chow diet (Envigo Teklad #2918) free of animal products or exogenous creatine to prevent cross contamination of measured creatine.

### 2.4 Body composition testing

Dual‐energy x‐ray absorptiometry (DXA) scans were used to quantify body composition in anesthetized (2% isoflurane) immobilized mice by InAlyzer2S (Micro Photonics). DXA scans were performed during the same week as D_3_‐Cr injections.

### 2.5 D_3_‐creatine dilution protocol

The day of the study, experimental mice were weighed before being administered a single dose of 2 mg/kg body weight D_3_‐creatine by intraperitoneal injection. Following the injection, mice were placed back into their cage with ad libitum access to food and water. Urine samples were collected 2, 6, 12, 24, and 48 h post‐dosing and stored at −80°C.

### 2.6 Assessment of urinary D3‐creatinine

Mouse urine was stored at −20°C until analyzed for D_3_‐creatine within the Nutrition Science and Toxicology Department / Hellerstein Laboratory at UC Berkeley. Urine samples were processed for D_3_‐creatinine enrichment, as well as creatine and creatinine concentrations to determine Cr/Crn ratio using methods described in detail elsewhere (Clark et al., 2014; Shankaran et al., 2018). Briefly, samples and standards were mixed with an internal standard of D5‐labeled Cr and Crn, proteins were precipitated by the addition of acetonitrile followed by centrifugation, and aliquots of the supernatant were diluted in acetonitrile for liquid‐chromatography mass‐spectrometry (LC/MS) analysis. Mass spectrometry was performed on a Sciex 6500 QTRAP (AB Sciex LLC, Framingham, MA) operating in multiple reaction monitoring mode. For determining the Cr and Crn concentration from which the Cr/Crn ratio was calculated, transitions (132.1/44.1) and (114.1/44.1) were monitored. Quantitation was performed using an external standard curve using an internal standard mixture of D5 labeled Cr and Crn following transitions (137.1/47.1) and (119.1/47.1). For determining the enrichment of D_3_‐Crn, quantitation was performed using a standard curve for D_3_‐Crn and D_3_‐Cr enrichment that spanned from 0% to 0.577% and measured multiple reaction monitoring transitions (114.1/44.1) corresponding to the M0 peak of Crn and 117.1/47.1 which corresponds to D3‐Crn.

### 2.7 Statistical analysis

Statistical analysis across all age groups was assessed by one‐way ANOVA, while unpaired analyses between specific age groups were assessed by t‐test. XY plot analyses were assessed by simple linear regression, and correlation values were calculated using Pearson's Correlation test. For gait analyses, XY plot analyses were assessed either by simple linear regression or second order polynomial (quadratic) nonlinear regression [Step cycle]. Significance is reported as follows: ns *p* > 0.05, *p < 0.05, **<0.005, ***<0.0005.

## AUTHOR CONTRIBUTIONS

SM, WE, and MH involved in conceptualization and supervision. LW, SS, MH, WE, and SM drafted the manuscript. LW, NE, and SS involved in analysis and visualization. EG, SG, EN, SS, AD, LP, MCM, and CP involved in experimental procedure. SE and NS provided operational support. All the authors have read and agreed to the final version of the manuscript.

## FUNDING INFORMATION

We acknowledge support from the Astera Institute and Ono Pharmaceuticals.

## CONFLICT OF INTEREST STATEMENT

The authors declare no conflicts of interest. Drs Hellerstein and Evans are listed as co‐inventors on the filed patents for the D_3_‐creatine dilution method; however, they do not derive any income related to commercial use of this method nor do they control the intellectual property.

## Supporting information


Figure S1

## Data Availability

The data that support the findings of this study are available from the corresponding author upon reasonable request.

## References

[acel13897-bib-0001] Kandutsch, A. A. , & Russell, A. E. (1958). Creatine and creatinine in tissues and urine of mice with hereditary muscular dystrophy. American Journal of Physiology, 194(3), 553–556. 13571426 10.1152/ajplegacy.1958.194.3.553

[acel13897-bib-0002] Alley, D. E. , Ferrucci, L. , Barbagallo, M. , Studenski, S. A. , & Harris, T. B. (2008). A research agenda: The changing relationship between body weight and health in aging. The Journals of Gerontology. Series A, Biological Sciences and Medical Sciences, 63(11), 1257–1259. 19038842 10.1093/gerona/63.11.1257 PMC4984841

[acel13897-bib-0003] Andrews, R. , Greenhaff, P. , Curtis, S. , Perry, A. , & Cowley, A. J. (1998). The effect of dietary creatine supplementation on skeletal muscle metabolism in congestive heart failure. European Heart Journal, 19(4), 617–622. 9597411 10.1053/euhj.1997.0767

[acel13897-bib-0004] Cawthon, P. M. , Blackwell, T. , Cummings, S. R. , Orwoll, E. S. , Duchowny, K. A. , Kado, D. M. , Stone, K. L. , Ensrud, K. E. , Cauley, J. A. , & Evans, W. J. (2021). Muscle mass assessed by the D3‐Creatine dilution method and incident self‐reported disability and mortality in a prospective observational study of community‐dwelling older men. The Journals of Gerontology. Series A, Biological Sciences and Medical Sciences, 76(1), 123–130. 32442245 10.1093/gerona/glaa111 PMC7756711

[acel13897-bib-0005] Cawthon, P. M. , Orwoll, E. S. , Peters, K. E. , Ensrud, K. E. , Cauley, J. A. , Kado, D. M. , Stefanick, M. L. , Shikany, J. M. , Strotmeyer, E. S. , Glynn, N. W. , Caserotti, P. , Shankaran, M. , Hellerstein, M. , Cummings, S. R. , Evans, W. J. , & Osteoporotic Fractures in Men (MrOS) Study Research Group . (2019). Strong relation between muscle mass determined by D3‐creatine dilution, physical performance, and incidence of falls and mobility limitations in a prospective cohort of older men. The Journals of Gerontology. Series A, Biological Sciences and Medical Sciences, 74(6), 844–852. 29897420 10.1093/gerona/gly129 PMC6521914

[acel13897-bib-0006] Cawthon, P. M. , Peters, K. E. , Cummings, S. R. , Orwoll, E. S. , Hoffman, A. R. , Ensrud, K. E. , Cauley, J. A. , Evans, W. J. , & the Osteoporotic Fractures in Men (MrOS) Study Research Group . (2022). Association between muscle mass determined by D(3) ‐Creatine dilution and incident fractures in a prospective cohort study of older men. Journal of Bone and Mineral Research, 37(7), 1213–1220. 35253257 10.1002/jbmr.4505 PMC9283198

[acel13897-bib-0007] Clark, R. V. , Walker, A. C. , O'Connor‐Semmes, R. L. , Leonard, M. S. , Miller, R. R. , Stimpson, S. A. , Turner, S. M. , Ravussin, E. , Cefalu, W. T. , Hellerstein, M. K. , & Evans, W. J. (2014). Total body skeletal muscle mass: Estimation by creatine (methyl‐d3) dilution in humans. Journal of Applied Physiology (Bethesda, MD: 1985), 116(12), 1605–1613. 24764133 10.1152/japplphysiol.00045.2014 PMC4064374

[acel13897-bib-0008] Evans, D. S. , O'Leary, M. N. , Murphy, R. , Schmidt, M. , Koenig, K. , Presley, M. , Garrett, B. , Kim, H. N. , Han, L. , Academia, E. C. , Laye, M. J. , Edgar, D. , Zambataro, C. A. , Barhydt, T. , Dewey, C. M. , Mayfield, J. , Wilson, J. , Alavez, S. , Lucanic, M. , … Melov, S. (2021). Longitudinal functional study of murine aging: A resource for future study designs. JBMR Plus, 5(3), e10466. 33778327 10.1002/jbm4.10466 PMC7990142

[acel13897-bib-0009] Giacomello, E. , Crea, E. , Torelli, L. , Bergamo, A. , Reggiani, C. , Sava, G. , & Toniolo, L. (2020). Age dependent modification of the metabolic profile of the tibialis anterior muscle Fibers in C57BL/6J mice. International Journal of Molecular Sciences., 21, 3923. 32486238 10.3390/ijms21113923 PMC7312486

[acel13897-bib-0010] Heymsfield, S. B. , Arteaga, C. , McManus, C. , Smith, J. , & Moffitt, S. (1983). Measurement of muscle mass in humans: Validity of the 24‐hour urinary creatinine method. The American Journal of Clinical Nutrition, 37(3), 478–494. 6829490 10.1093/ajcn/37.3.478

[acel13897-bib-0011] Meganck, J. A. , & Liu, B. (2017). Dosimetry in micro‐computed tomography: A review of the measurement methods, impacts, and characterization of the quantum GX imaging system. Molecular Imaging and Biology, 19(4), 499–511. 27957647 10.1007/s11307-016-1026-x PMC5498628

[acel13897-bib-0012] Pappas, L. E. , & Nagy, T. R. (2019). The translation of age‐related body composition findings from rodents to humans. European Journal of Clinical Nutrition, 73(2), 172–178. 30283153 10.1038/s41430-018-0324-6 PMC6620047

[acel13897-bib-0013] Pirker, W. , & Katzenschlager, R. (2017). Gait disorders in adults and the elderly: A clinical guide. Wiener Klinische Wochenschrift, 129(3–4), 81–95. 27770207 10.1007/s00508-016-1096-4 PMC5318488

[acel13897-bib-0014] Schaap, L. A. , Koster, A. , & Visser, M. (2013). Adiposity, muscle mass, and muscle strength in relation to functional decline in older persons. Epidemiologic Reviews, 35, 51–65. 23221972 10.1093/epirev/mxs006

[acel13897-bib-0015] Shankaran, M. , Czerwieniec, G. , Fessler, C. , Wong, P. Y. A. , Killion, S. , Turner, S. M. , Hellerstein, M. K. , & Evans, W. J. (2018). Dilution of oral D(3) ‐Creatine to measure creatine pool size and estimate skeletal muscle mass: Development of a correction algorithm. Journal of Cachexia, Sarcopenia and Muscle, 9(3), 540–546. 29663711 10.1002/jcsm.12278 PMC5989770

[acel13897-bib-0016] Shepherd, J. A. , Ng, B. K. , Sommer, M. J. , & Heymsfield, S. B. (2017). Body composition by DXA. Bone, 104, 101–105. 28625918 10.1016/j.bone.2017.06.010 PMC5659281

[acel13897-bib-0017] Stimpson, S. A. , Turner, S. M. , Clifton, L. G. , Poole, J. C. , Mohammed, H. A. , Shearer, T. W. , Waitt, G. M. , Hagerty, L. L. , Remlinger, K. S. , Hellerstein, M. K. , & Evans, W. J. (2012). Total‐body creatine pool size and skeletal muscle mass determination by creatine‐(methyl‐D3) dilution in rats. Journal of Applied Physiology (Bethesda, MD: 1985), 112(11), 1940–1948. 22422801 10.1152/japplphysiol.00122.2012

[acel13897-bib-0018] Volpi, E. , Nazemi, R. , & Fujita, S. (2004). Muscle tissue changes with aging. Current Opinion in Clinical Nutrition and Metabolic Care, 7(4), 405–410. 15192443 10.1097/01.mco.0000134362.76653.b2 PMC2804956

[acel13897-bib-0019] Wang, Z. M. , Pierson, R. N., Jr. , & Heymsfield, S. B. (1992). The five‐level model: A new approach to organizing body‐composition research. The American Journal of Clinical Nutrition, 56(1), 19–28. 1609756 10.1093/ajcn/56.1.19

