# Correction to: The D3‐creatine dilution method non‐invasively measures muscle mass in mice

**DOI:** 10.1111/acel.13999

**Published:** 2023-10-02

Wimer, L., Goncharova, E., Galkina, S., Nyangau, E., Shankaran, M., Davis, A., Prado, L., Munoz, M. C., Epstein, S., Patterson, C., Shaum, N., Hellerstein, M., Evans, W., & Melov, S. (2023). The D3‐creatine dilution method non‐invasively measures muscle mass in mice. *Aging Cell*, 22, e13897. https://doi.org/10.1111/acel.13897

The author's name was misspelled as Nicholas Shaum. This should have read as Nicholas Schaum.

We apologize for this error.

