# Supplementary material for: The D3 ‐creatine dilution method non‐invasively measures muscle mass in mice
Source: Aging Cell. 2023 Jun 5;22(8):e13897. doi: 10.1111/acel.13897 (PMC10410001; doi:10.1111/acel.13897)
Supplement: Supplementary file 1 — Figure S1 [file ACEL-22-e13897-s001.docx]

**Figure S1: D_3_-Cr dilution method determined muscle weights compared to DXA-determined lean muscle mass, body weights, and dissected hindlimb muscle weights.** Correlations of DXA-determined lean body mass (LBM) to their respective D3-Cr dilution method determined muscle mass (D3-Crn MM) in the cross-sectional study grouped A) together and B-C) separated by sex. D) Correlations of both DXA-LBM and D3-Cr MM with their respective body weights from the pilot study described in Figure 1. E) Dissected hindlimb muscle weights of mice from the cross-sectional aging study described in Figure 2.
